# Application of Multiomics in Perinatology: A Metabolomics Integration-Focused Review

**DOI:** 10.3390/ijms26094164

**Published:** 2025-04-27

**Authors:** Alice Bosco, Francesca Arru, Alessandra Abis, Vassilios Fanos, Angelica Dessì

**Affiliations:** Neonatal Intensive Care Unit, Department of Surgical Sciences, University of Cagliari, AOU Cagliari, 09124 Cagliari, Italy; alicebosco88@gmail.com (A.B.); f.arru7@studenti.unica.it (F.A.); a.abis17@studenti.unica.it (A.A.); vafanos@tiscali.it (V.F.)

**Keywords:** multiomics, perinatology, perinatal metabolomics, precision medicine, high-throughput technologies

## Abstract

Precision medicine stems from a new approach to the prevention, diagnosis and treatment of patients, due to the shift in focus away from pathology and towards the uniqueness of the individual, personalising the diagnostic–therapeutic pathway. This paradigm shift has been made possible by the emergence of new high-throughput technologies capable of generating large amounts of data on multiple levels of a biological system, identifying pathology-related genes, transcripts, proteins and metabolites. Metabolomics plays a primary role in this context, providing, through non-invasive sampling, a very close image of the phenotype of the organism being studied by detecting metabolites, end products downstream of gene transcription, present in cells, tissues, organs and biological fluids. The enormous amount of data that these modern technologies make available, together with the need to elucidate the complex interplay of the various biological levels by combining data from distinct omics, has led to the need to employ advanced informatics techniques, among which artificial intelligence has recently emerged. These innovations are of great interest in the field of perinatology, representing an attempt to optimise the diagnostic timeline for the most critical newborns. In addition, they may contribute to the improvement of prevention strategies available to date. All these contributions prove to be crucial at very vulnerable life stages, allowing crucial intervention opportunities. In this review, we have analysed studies that have integrated metabolomics with at least one other omics in the perinatal field, attempting to highlight the usefulness of multiomics integration and the different methods employed.

## 1. Introduction

In recent years, the world of medicine has been undergoing a major change, shifting from evidence-based treatment protocols focused on the average patient to care tailored to an individual’s peculiarities. This transition is possible thanks to the emergence of precision medicine, which, through the integration of multiomics data with medical history, individual choices, and environmental knowledge, allows for the precise characterisation of health and disease states, along with optimal treatment options [1].

Thus, the integration of precision medicine into health care can have a very important impact on public health through more accurate and earlier diagnosis, through an important preventive intervention that can predict the risk of disease before symptoms occur, and through the design of personalised treatment plans characterised by high standards of safety and efficiency [1].

To date, genotype-driven treatment is the most studied impact of precision medicine on health care, particularly in optimising drug therapies [2] and creating targeted therapy plans for certain cancer patients (breast and lung cancer) [3]. Precision medicine relies on various data collection and analysis technologies that provide specific phenotypes, often integrated with electronic health records (EHRs) [1].

Advances in high-throughput technologies have enabled the generation of large amounts of data at multiple levels of a biological system, identifying genes (genomics), transcripts (transcriptomics), proteins (proteomics), and metabolites (metabolomics) associated with specific diseases or phenotypes of interest. Such associations may represent valuable biomarkers and help elucidate complex underlying pathogenetic mechanisms. However, it has soon become evident that single-omics studies struggle to provide the complex interplay of various biological levels, thus necessitating multiomics studies that integrate data from different biological domains. This synergy of studies can provide a more detailed molecular understanding of health and disease in order to identify increasingly effective therapies. However, identifying complex relationships among multiomics data requires significant statistical re-processing of the data along with a machine learning (ML) approach, including Bayesian networks, pathway enrichment analysis, partial least squares analysis and many others [4,5,6,7,8].

In this approach, metabolomics and lipidomics, a branch of metabolomics that deals with lipid metabolites, play a unique role in providing a detailed picture of an organism’s phenotype by capturing biochemical responses to stimuli through metabolite analysis. These low molecular weight molecules represent the end products of gene expression and are present in cells, tissues, organs, and biological fluids. Their detection also allows for untargeted investigations, free from prior assumptions and independent of existing knowledge and results. This results in an in-depth study of disease pathogenesis, selection of individuals at higher risk, with a view to predictive medicine, and early differential diagnosis through identification of biomarkers with high diagnostic power together with minimally invasive sampling, with the ultimate goal of providing appropriate and specific treatment [9,10]. Indeed, growing evidence from large-scale epidemiological studies supports the increasing role of metabolomics in integrative analysis [4].

Another important building block of precision medicine is the creation of data repositories, biobanks, which are crucial to address a further phase of the healthcare revolution: the integration of precision medicine with artificial intelligence (AI). This would optimise both public and private economic investments because it would increase the scientific reproducibility of metabolomics and other omics sciences [1]. Several public data repositories are available for metabolomics, the most important of which are Metabolomics Workbench of the National Institutes of Health (NIH) and MetaboLights of the European Bioinformatics Institute (EBI), part of the European Molecular Biology Laboratory (EMBL) [11,12].

These advancements directly involve perinatology, not only in enabling timely diagnoses for critically ill newborns (e.g., premature, necrotising enterocolitis, perinatal asphyxia) but also in identifying and monitoring those at higher risk of developing disease (e.g., IUGR, children of diabetic mothers) [9,13,14]. It is well established that the environment experienced during the early years of life has a significant impact on subsequent health and development. Evidence suggests that even in utero, adaptive responses to environmental cues can predict later health outcomes and mismatches between the prenatal and postnatal environment may underlie the fetal and infant predispositions to important noncommunicable diseases, such as obesity, cardiovascular disease, and other metabolic alterations [15].

In this review, we aimed to investigate the state of the art on the application of multiomics studies in perinatology, evaluating their contributions, limitations, and potential, focusing on the integration of metabolomic (and lipidomic) data with one or more other omics data.

## 2. Multiomics Integration: From Spearman Correlation Analysis to Artificial Intelligence

Future medicine must focus on a deeper understanding of the biological systems and the molecular mechanisms underlying disease development. This requires integrating various omics disciplines, including transcriptomics, proteomics, microbiomics, and metabolomics. Omics data help identify various differential factors potentially associated with disease, which also serve as biomarkers of the disease itself. However, single-omics analysis provides only partial insights, whereas multiomics integration, i.e., combining information from multiple omics levels, provides a deeper understanding of the disease process, thereby clarifying potential causal changes, helping to capture potential therapeutic targets [16].

Advances in these high-throughput technologies have enabled the simultaneous processing of vast datasets. However, the collective quantification and characterisation of such large amounts of biological data have required many statistical integration efforts, necessitating the application of advanced computer techniques [4,16,17,18,19,20,21,22,23].

A recent review of the literature on multiomics integration methodologies showed that, to date, various data integration methods have been applied in multiomics analyses conducted. Frequently observed are more conventional methods, such as Spearman’s and Pearson’s correlation analysis, or generalised linear models (GLM), including logistic regression, Poisson distribution and negative binomial regression. There are also machine learning (ML) methods, such as random forest (RF) and support vector machine (SVM). However, conducting integrative statistical analyses of large-scale biological data in an attempt to identify significant factors and possibly combine them has often been pursued by analysing the same type of data from different sources or different types of data from the same source. This leads to a high risk for this approach of failing to identify both linearly and non-linearly associated multiomic interactions [16].

Certainly, important advances have involved software tools for the integration of multiple omics datasets, based on pathway enrichment, biological network and empirical correlation analysis. However, the methods have limitations; pathway enrichment analysis relies solely on predefined pathways, far removed from the complexity of biological systems, while biological network analysis requires extensive domain knowledge about interactions between genes, proteins and metabolites, which is not always available. Empirical correlation analysis, although easy to implement, often lacks deep insight and is often replaced by more sophisticated approaches. These include Bayesian networks, which, although employed even at low sample sizes, are limited by the need for extensive prior knowledge to estimate probabilistic interactions between modelled variables [24].

To address these limitations, advanced ML-based multiomics approaches have become popular [16]. ML, a subset of AI, uses algorithms that build predictive models based on observed data [20,21,22,23]. The distinction between AI and other forms of technology lies in its capacity to utilise algorithms and models that enable machines to undertake tasks that would otherwise require human intelligence [15]. On the other hand, ML refers to the input of data into a machine that, through an algorithm, can learn without being explicitly programmed. However, other authors consider ML simply as the main application of AI [21].

These modern analytical technologies are able to exploit the synergy of multiomics information by identifying the complex interactions between such data, using network analysis, with the goal of achieving more accurate prediction of clinical outcomes. In addition, the integration of multiomics data with ML enables high-level biological representation of canonical variables in multiomics data using matrix factorisation, principal component analysis (PCA), Partial Least Squares Discriminant Analysis (PLS-DA) regression and canonical correlation. This allows the identification of different disease subtypes through clustering and classification methods [16,17,24]. This approach, thus, provides a more holistic view of disease pathogenesis, with the goal of identifying specific diagnostic biomarkers and developing predictive models suitable for identifying risk before the condition is clinically evident, with a view to optimising diagnostic timing [16].

Among the ML algorithms already commonly applied to metabolomic data only for classification and regression analysis are RF, an algorithm based on decision tree theory, with good performance for high dimensionality data and excellent adaptability to unbalanced and missing values. Additionally, SVM is useful in separating data with N data points into (N − 1) dimensional hyperplanes, and linear discriminant analysis, closely related to analysis of variance and regression analysis, is useful for expressing a dependent variable as a linear combination of other characteristics or measures. Another method is prediction analysis for microarrays (PAM), which classifies different samples from gene expression data. Furthermore, the generalised linear model, including the Least Shrinkage and Selection Operator (LASSO), is particularly useful for variable selection and dimensionality reduction. Notably, among generalised linear models, logistic regression is particularly useful for assessing the relationship between the categorical dependent variable and one or more independent variables by estimating the probabilities using a logistic function, namely the cumulative logistic distribution [16,24].

Recently, advancements in ML algorithms have led to deep learning (DL), also known as deep neural network or artificial neural network (ANN). As an evolution of ML, DL can be considered a further advance in AI [22]. It relies on multilayer neural networks that learn from a large volume of data, both in a supervised and unsupervised manner [16,21,22]. Unlike conventional ML methods, where there is human input of specific features, DL is distinguished by a mode of learning representation directly from raw data, even images [20]. This offers a twofold advantage: it allows both independent analysis of each type of multiomics data and the integration of different data levels, spanning multiomics as well as medical or health records with high sensitivity, specificity and efficiency. DL is considered a self-taught AI method, not based on fixed mathematical formulas but rather on multiple hidden layers. The depth of these layers determines the ability to learn complex models and influences the accuracy of predictions [16]. However, despite the advantages of DL over conventional ML methods in handling high-volume, high-dimensional data, some DL processing layers are hidden from the human user, leading to increased complexity in interpretation and raising concerns regarding model accountability [20].

**Figure 1:**
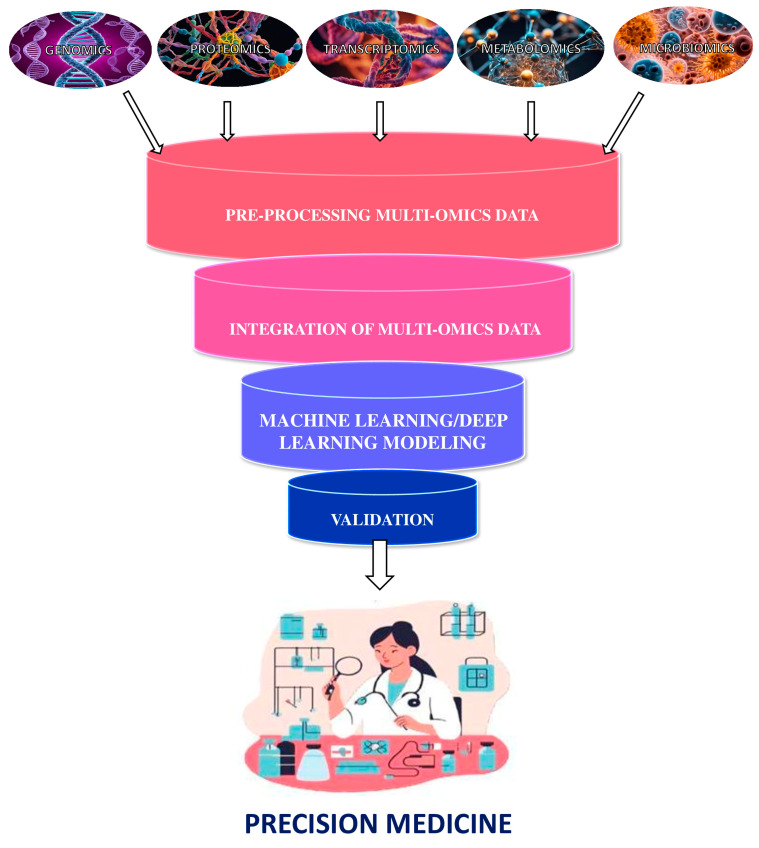
The synergy between modern high-throughput technologies and computer science for precision medicine.

The basis of precision medicine, built on the synergy between modern high-throughput technologies and computational science is schematized in Figure 1.

## 3. Multiomics Studies in Perinatology

### 3.1. Pregnancy

Multiomics studies involving the use of metabolomics along with at least one other omics have found greatest application in major obstetric syndromes, such as preeclampsia (PE), intrauterine growth restriction (IUGR), gestational diabetes (GDM), and spontaneous preterm delivery (sPTD) [25,26,27,28,29,30,31,32,33,34,35,36,37,38,39,40,41]. Underlying these common obstetric syndromes is frequently placental dysfunction, evidenced by similar histopathological patterns of the placenta, although the molecular alterations that characterise patients within each syndrome are different. This results in a difficulty in preventing and treating these conditions, which result in millions of perinatal and infant deaths each year globally, with a higher risk in males [25,26].

Table 1 summarizes the multiomics studies on major obstetrical issues that integrated metabolomic data with one or more other omics data also highlighting the type of correlation analysis performed between the different omics, where present in the studies.

Gong et al. [25], analysed the placental transcriptome and serum metabolome of 4212 women in their first pregnancy who were part of the Pregnancy Outcome Prediction (POP) study. The results of this analysis demonstrated that the synthesis of placental polyamines differs between the sexes. Specifically, it was evidenced that spermine synthase (SMS) evades X-chromosome inactivation (XCI) and is expressed at lower levels in male primary trophoblastic cells. Additionally, the spermine metabolite N1,N12-diacetylspermine (DiAcSpm) was identified as being higher in the female placenta and serum of women with female fetuses. It was also observed that this pathway is implicated in the pathophysiology of pregnancy complications related to placental dysfunction. In fact, higher maternal serum levels of DiAcSpm were found to be associated with an increased risk of PE but a decreased risk of IUGR, placing this metabolite as the first maternal biomarker having opposite associations with PE and IUGR. For the metabolite analysis at 36 weeks, the study employed *p* < 0.01 without FDR correction, while for the other analyses (e.g., gene expression), false discovery rate (FDR) control was performed, with multiple test correction, using the Benjamini–Hochberg procedure [25].

A similar study was conducted by Barak et al. [26] using data from a biobank (more than 300 samples subjected to multiomics analysis). Through an artificial intelligence-based similarity network fusion (SNF), they integrated different data and placental clusters defined by various omics (including transcriptomics, proteomics and metabolomics), together with histological evaluation, to study the placental dysfunctions underlying the most common obstetric syndromes (PE, IUGR, sPTD). In fact, it is known that although these issues share similar histopathological features, they manifest themselves with different molecular alterations, complicating treatment. The results revealed four distinct clusters, each associated mainly with an obstetric syndrome, in contrast to the conventional pairwise comparison. Specifically, an early-onset PE-dominated cluster was revealed that showed strong patterns of placental dysfunction while minor patterns of injury were detected in the sPTD-dominated cluster. In addition, through omics integration, a better correlation with histopathology could be observed than in the predefined disease clusters. Furthermore, the main metabolic alterations highlighted, especially in FGR and HDP, included mainly lipids (sphingolipids) and amino acids. Metabolic pathway analysis also showed involvement in interleukin signalling, interferon and glucose metabolism. Therefore, the authors concluded that the integration of data from the different omics allows for a distinct reclassification of placental dysfunction patterns underlying common obstetric syndromes that can facilitate a better understanding of disease processes in an effort to implement increasingly personalised and effective interventions. In the differential expression analysis of the omics data, in order to ensure the necessary statistical rigour, multiple test correction was applied using the Benjamini–Hochberg procedure, checking the FDR [26].

A PE risk prediction model based on the analysis of six omics datasets from a longitudinal cohort of pregnant women was developed by Marić et al. [27]. The challenging goal of this study was to conduct an integrated analysis of a large high-throughput omics dataset (proteome, transcriptome, plasma and urinary metabolome, lipidome, and vaginal microbiome) containing more than 50,000 measurements per sample. This machine-learning (ML)-based multiomics model showed high accuracy (area under the receiver operating characteristic curve, AUC, of 0.94, 95% confidence intervals (CI) [0.90, 0.99], supporting that a uniform comparison of multiple omics datasets exhibits better predictive ability of PE status than individual modes of analysis. Nevertheless, good prediction accuracy, both during gestation and early pregnancy, was also observed for plasma proteomic and urine metabolomic datasets. In fact, a prediction model with an AUC of 0.87 (95% CI [0.76, 0.99]) was validated using an independent cohort of 16 women, which, using only 10 urinary metabolites, provided an accuracy equal to that of the entire metabolomic dataset. The main metabolic alterations observed included tryptophan metabolism involving kynurenine pathways, fatty acid and arachidonic acid metabolism, together with steroid hormone metabolism. These results led the authors to conclude that this research represents the basis for the design of a simple early diagnostic test for PE, after validation in larger populations. Also, in this case, the Benjamini–Hochberg procedure was applied for check the FDR [27].

In order to evaluate the effectiveness of metabolomics in predicting PE and its integration with transcriptomics, Kelly et al. [28] conducted a prospective study on a cohort (47 PE and 62 controls) of patients from the VADAART (multicentre, randomised, double-blind, placebo-controlled clinical trial). The results showed that plasma metabolites were already present in the first trimester of pregnancy and were capable of predicting the risk of subsequent PE in the third trimester. The metabolites involved were associated with a lipid imbalance, a feature confirmed and deepened through integration with transcriptomic data, with the greatest alterations observed in the metabolism of glycerophospholipids, arachidonic acid, glycerolipids and fatty acid biosynthesis. The important role of lipids in immune function emerged, highlighting their centrality in the pathogenesis of PE. Integration between metabolomic and transcriptomic data was performed using Weighted Gene Correlation Network Analysis (WGCNA), a correlation clustering technique. However, the authors report that despite the absence of a formal correction for multiple testing, some metabolites and biological pathways were confirmed through replication between first- and third-trimester samples, supporting some degree of biological validation [28].

Odenkirk et al. [29] conducted a multiomics (proteomics and lipidomics) analysis using multidimensional analysis methods and cheminformatic visualisation tools to elucidate the molecular changes occurring in patients with PE and gestational diabetes mellitus (GDM). First, a separate analysis of each omics was conducted, and then the structural and biological relationships of the identified molecules were analysed, which highlighted proteins capable of establishing associations with lipids. The results of the proteomics and lipidomics were then combined in a multiomics comparison analysis, which illustrated the unique molecular mechanisms of each disease. Indeed, several direct protein–lipid associations were revealed from the multiomics analysis supporting a correlation between protein and lipid dysregulation. Specifically, in the case of GDM, lipid species plasmalogen phosphatidylethanolamine (PE P) with 22:6 acyl groups were found to be up-regulated, while phosphatidylethanolamine binding proteins-1 (PEBP1), which preferentially binds to phosphatidylethanolamines (PE) was down-regulated. This association was significant only in GDM. In contrast, in PE, both phosphatidylinositol–glycan lipid species and the phosphatidylinositol–glycan-specific phospholipase D (PHLD) enzyme were found to be consistently downregulated. Again, the association observed was unique to PE. Therefore, the authors of the paper concluded that through the multiomics approach, a direct relationship between proteins and lipids could be evidenced, suggesting a strong interdependence in the origin and progression of the two diseases under analysis, thus providing a deeper understanding of the molecular profiles of the end stage of PE and GDM. They also envisioned the use of these alterations as possible biomarkers for early diagnosis of GDM and PE, although these observations need to be validated with longitudinal assessment throughout gestation in order to identify early molecular changes prior to clinical diagnosis. Multiple test correction was performed through analysis of variance (ANOVA) with Dunnett’s correction and g-test with Bonferroni’s correction. Adjusted *p*-values ≤ 0.05 were considered to ensure statistical significance for protein and lipids [29].

Another multiomics study that investigated the placental lipid profile in GDM was conducted by Easton et al. [30]. They integrated transcriptomics (mRNA microarrays), metabolomics data through a pathway enrichment analysis software tool (MetaboAnalyst v5), to analyse the impact of two non-esterified fatty acids (NEFAs), palmitate (PA) and oleate (OA), on placental villous trophoblast cell metabolism through the use of the BeWo cell line model. Indeed, it is now known that these fatty acids, the most abundantly consumed in the diets of Westernised populations, are potential independent regulators of placental lipid management and, furthermore, elevated fasting serum levels of these lipid species characterise women with GDM and obesity. Multiomics analysis showed that exposure to PAs is associated with enrichment in β-oxidation pathways, while exposure to OAs is associated with enrichment in anti-inflammatory and antioxidant pathways. These observations led the authors to conclude about the importance of appropriate dietary advice and specific nutritional interventions to maintain adequate placental function. In this study, the analysis of metabolic pathways was based on raw *p*-values, without reference to FDR or other corrections for multiple testing [30].

Another multiomics study on GDM-related alterations was conducted by Dong et al. [31], who conducted 16S rRNA sequencing of the faecal microbiota and H1-NMR profiling of the plasma metabolome of 20 women with GDM and 20 nondiabetic controls. Multiomics correlation (co-inertia analysis, procrustes analysis and redundancy analysis) revealed a close correlation between the faecal microbiota and plasma metabolome in GDM related to five plasma metabolites (glycerol, lactic acid, proline, galactitol and methylmalonic acid) and 98 members of the faecal microbiota. To further evaluate the correlations of GDM-associated clinical indices with metabolites contributing to alterations in the faecal microbiota, Spearman rank correlation analysis was performed, which showed that four of the five metabolites (except galactitol) were correlated with hyperglycemia. In contrast, co-occurring network analysis showed that 15 of the 98 members of the faecal microbiota were positively correlated with each other, forming a co-occurring cohort (mainly consisting of the phylum Firmicutes). The authors concluded that the multiomics approach highlighted new therapeutic strategies for GDM targeting the gut microbiota. FDR control for multiple test correction was performed for both metabolomic and microbiome data, with a significance threshold of *p* < 0.05 after correction [31].

Liu et al. [32] also conducted a multiomics analysis inherent in the correlation between gut microbiota and metabolome with the aim of investigating their potential transmission to the fetus. With a prospective cohort study, they analysed 89 mother–infant pairs (44 in the GDM group and 45 in the normoglycemic group) through 16S rRNA gene sequencing and nontargeted plasma metabolomics, implemented by integrative analysis (Spearman correlation analysis) to elucidate the interactions between these data. GDM mothers were characterised by different microbial and metabolic signatures compared to the control group. In particular, the analysis showed a different impact on metabolites related to glucose, lipid and energy metabolism in the GDM group. With regard to the main altered metabolites in the study were the glycerophospholipids (e.g., PC(18:0/20:3(5Z,8Z,11Z)), which were significantly decreased in both mothers and infants with GDM compared to controls. An opposite trend emerged for certain polyunsaturated fatty acids (e.g., PE (18:4/22:6)), which were found to be decreased in diabetic mothers while they were increased in GDM infants. An alteration in bile acid metabolism, certain lipid metabolites related to the metabolic pathway of unsaturated fatty acids and lipid biosynthesis was also observed in both mothers and diabetic offspring. Correlation analysis (Spearman correlation) also showed a dual influence of the microbiota on metabolic homeostasis, a direct action and one through modulation of metabolites. These findings further support the importance of multiomics analysis in studying the mechanisms underlying the transmission of metabolic alterations across generations. Multiple test correction was applied using the Benjamini–Hochberg procedure with differences considered statistically significant for FDR-adjusted values below 0.05) for both metabolomic and microbiomic data [32].

A correlation between the intestinal mycobiome during pregnancy and GDM was observed in the study by Fu et al. [33]. They analysed the intestinal mycobiome of pregnant women in an attempt to reveal possible associations with the metabolome and the course of pregnancy itself. The results of the study showed significant interactions between intestinal mycobiome, biological function, serum metabolites and pregnancy course. Specifically, correlation analyses between individual genera of the fungal core and individual metabolites showed 30 covariant relationships inherent in six genera and 27 serum metabolites. Correlation analysis between the fungal enterotypes and individual functional pathways of the gut microbiome identified eight pathways whose distribution varied between enterotypes, and through regression analysis, 95 significant associations between the identified pathways and 27 serum metabolites were revealed. In particular, the correlation between mycobiome and serum metabolome showed that intestinal fungi are potential modulators of specific metabolic pathways, such as those associated with fatty acids and amino acids. The existence of a correlation between the presence of Mucor and the onset of gestational diabetes mellitus/fetal macrosomia was also shown. FDR control was performed, with multiple test correction [33].

Tao et al. [34], on the other hand, analysed the relationship between the gut microbiome and metabolome as a function of fetal growth, specifically in IUGR. An analysis of the gut microbiome, faecal and serum metabolome was conducted in 70 pregnant women (35 IUGR and 35 controls) by integrating the multidimensional data to reveal links between the datasets. With a Spearman’s correlation coefficient of 0.3, a significant association was shown between 25 major bacterial species and 13 serum metabolites (allantoin, pinitol, nicotinic acid, malic acid, phosphatidic lysoacid, maltose, 9-hexa-decene, glycol-diacid-2-phosphate) together with four of faecal origin (dodecanoic glycol, dodecanoic glycol IV and pyrraline). A faecal microbiota transplantation mouse model was also employed to study the effects of the gut microbiome on fetal growth and placental phenotypes. Overall, the integration of the microbiome and metabolite profiles of the human cohort showed that patients with IUGR have gut dysbiosis and metabolic disorders, which contribute to the pathogenesis of the disease, also suggesting specific interactions not only between gut microbiota and metabolites but also with clinical measures. In detail, a group of microbial species altered in IUGR correlated closely with fetal measures and maternal clinical variables, and both faecal and serum metabolites altered in IUGR were also found to be associated with specific clinical phenotypes. In the faecal transplantation of the microbiota of women with IUGR in the mouse model, however, it was observed that such dysbiosis induced growth retardation and placental dysfunction, including altered remodelling of the spiral artery and insufficient invasion of trophoblast cells. Both metabolomic and microbiome data were corrected by FDR for multiple testing. Specifically, for the microbiota analysis, the *p*-value was adjusted with FDR for differential bacterial diversity and abundance analyses with different significance thresholds (*p* < 0.05, *p* < 0.01 and *p* < 0.001). Also in the metabolomic analysis, FDR was applied to correct *p*-values and an adjusted *p*-value < 0.001 was considered significant for differences in serum metabolites [34].

Another multiomics cohort study on IUGR with a small sample of subjects (20 pregnant women, nine IUGR and 11 controls) was conducted by Tang et al. [35] in an attempt to investigate the potential relationships between mRNA, gut microbiota, and metabolism underlying the pathology. Cord blood, maternal serum, stool, and placental tissue samples were collected, to which RNA sequencing, 16S rRNA sequencing, and metabolomics methods were applied and revealed several alterations. Through Spearman correlation analysis, the correlation between differential circulating mRNAs, microbiota, and metabolites was investigated, bringing out how some altered metabolites, including methionine and alanine, changes in some microbial species (*Tyzzerella*) and alteration of some circulating mRNAs (TRIM34, SMOX, FAM83A, NAPG) could represent mediators in the interaction between the gut and the circulatory system in IUGR. To ensure a statistically significant correlation when analysing differential mRNA, microbiota and metabolites, Spearman’s correlation coefficient was used, with an adjusted *p*-value considered significant if *p* < 0.05, without FDR correction [35].

Other common obstetric syndromes investigated with a multiomics approach include sPTD. In addition to what has already been discussed regarding the contribution of integrating data from different omics in reclassifying more clearly the patterns of placental dysfunction underlying sPTD [26], other studies have opted for other types of omics integration [36,37]. Jehan et al. [36], with the support of the Alliance for Maternal and Newborn Health Improvement and from the biorepositories of the Global Alliance to Prevent Prematurity and Stillbirth, wanted to test the ability of plasma transcriptomics and proteomics and urine metabolomics analysis to identify early biological measures associated with PTD, with a view to making generalisable biological models for more timely diagnosis. Pregnant women from five cohorts in low- and middle-income countries (LMIC; i.e., Matlab, Bangladesh; Lusaka, Zambia; Sylhet, Bangladesh; Karachi, Pakistan; and Pemba, Tanzania) were analysed. Using a higher-level ML model, the results of the different datasets in the distinct cohorts were combined into a final integrative model that showed higher accuracy, with an area under the receiver operating characteristic curve (AUROC) of 0.83 (95% CI, 0.72–0.91), than the independent biological mode models. An inflammatory component (mainly from proteomic and transcriptomic data) and specific alterations in the urinary metabolome related to glutamine and glutamate metabolism and the valine, leucine, and isoleucine biosynthesis pathways were found to be the main features associated with this issue. This led the authors to postulate that in low-income countries and in settings with high PTD, key biological adaptations during term pregnancy follow a generalisable pattern. An increase in predictive accuracy for PTD correlated with the use of a multiomics approach, supporting its applicability for the development of predictive tests. Multiple test correction was performed through Bonferroni (*p* < 0.05) corrections for transcriptomic and FDR (*p* < 1.0 × 10^−6^, while some tests report incorrect *p* values because they are more specific or perform limited comparisons for proteomics analysis. A correction for multiple testing is not explicitly mentioned for metabolomic analysis, although Fisher’s tests for enrichment of metabolic pathways were used with very stringent significance thresholds (*p* < 4.4 × 10^−9^ for glutamine and glutamate metabolism, *p* < 7.3 × 10^−6^ for valine, leucine and isoleucine biosynthesis) [36].

A contribution in providing an integrated view of epidemiological factors related to PTD comes from the work of Espinosa et al. [37]. They conducted multiomics profiling by analysing plasma samples from 231 pregnant women to generate proteomic, metabolomic, and lipidomic datasets that revealed specific biological signatures of clinical covariates influencing this disease. Indeed, through ML models, robust performance emerged for the prediction of PTD (AUROC = 0.70), time to delivery (r = 0.65), maternal age (r = 0.59), gravidity (gravida pass me the term so much then it gets translated) (r = 0.56) and BMI (r = 0.81). In detail, specific fetal proteins (e.g., ALPP, AFP, and PGF) and immune proteins (e.g., PD-L1, CCL28, and LIFR) showed correlation with time to delivery, while collagen COL9A1 correlated with maternal age, endothelial NOS and inflammatory chemokine CXCL13 with gravidity, and leptin and structural protein FABP4 with BMI. The correction for multiple testing was performed using Bonferroni’s method. Spearman’s correlation was used to quantify the strength of associations between multiomics characteristics and only correlations with Bonferroni-adjusted *p*-values of <0.05 and |Rho| > 0.38 were considered significant [37].

A very recent work was conducted by Wu et al. [38] to analyse, through a multiomics analysis with RNA sequencing, nontargeted metabolomics, and targeted lipidomics, the phenotype and function of placental mucosa-associated invariant T cells (MAITs) and their specific mechanisms in PTD. The results first showed aberrant immune activation and abnormal increase of lipids and lipid-like metabolites in the placental microenvironment in PTD. In addition, heatmap correlation analysis showed that placental MAIT cell activation and expression of pro-inflammatory tissue molecules were positively correlated with the levels of the most elevated metabolites (sphingolipids, lysolipids, acylcarnitines). These data led the authors to conclude that in PTD, the altered activation and function of MAIT cells (decreased cytotoxicity and increased immunosuppression) may be related to the abnormal increase in lipids and lipid-like metabolites. The correction method for multiple tests is not explicitly specified in the study but *p*-value < 0.05 was used to ensure statistical significance [38].

Given the clinical importance of estimates regarding the timing of delivery and the scarce data currently available on the subject, Ghaemi et al. [39] conducted a multiomics analysis (immunome, transcriptome, microbiome, proteome, and metabolome) to understand the changes in the chronology of these systems during full-term pregnancy to provide the basis for the study of deviations implicated in pregnancy-related pathologies, including PTD and PE. Different levels of ML were applied. A first level, through a multivariate predictive model based on the Elastic Net (EN) algorithm, allowed the measurement of the predictive ability of gestational age of each dataset, with plasma proteomics showing the highest predictive power among the individual datasets. A further level of ML, through stacked generalisation, combined these datasets into a single model that significantly increased the predictive power by also revealing new interactions among the multiomics datasets. These include the correlation between pregnanolone sulfate and the immune system, between the oral microbiome and the TCRγδ⁺ cells, important regulators of mucosal immune response. Multiple test correction was performed through Bonferroni corrections with the significance threshold adjusted according to the number of comparisons made in the dataset [39].

The same goals inspired the work of Stelzer et al. [40], who also included preterm infants in their observational study, albeit in small numbers. They longitudinally analysed 63 women who had spontaneous labour in order to comprehensively characterise the maternal biology preceding labour in an attempt to identify biomarkers predictive of delivery. Important transitions in the feto–maternal immune, metabolic and endocrine systems characterised the transition of pregnancy to labour. Serial blood samples collected during the last 100 days of pregnancy were analysed for more than 7000 plasma analytes and peripheral immune cell responses using untargeted mass spectrometry, aptamer-based proteomic technology and single-cell mass cytometry. A multiomics model integrated all combined metabolomic, proteomic and immunomic datasets (stacked generalisation method) that provided an accurate prediction of the time of labour, independent of gestational age, based on estimated delivery date in both term and preterm births. Indeed, a molecular transition from maintained pregnancy to preterm biology was observed 2 to 4 weeks before delivery and was characterised by specific coordinated alterations in the metabolome, proteome and maternal immunome. The main alterations observed involved an increase in steroid hormone metabolites and interleukin-1 type 4 receptor before labour, coinciding with the shift from immune activation to regulation of inflammatory responses. Thus, the authors concluded that these findings represent the starting point for the design of new types of blood investigations to predict the day of labour based on shared mechanisms in preterm and term pregnancies. FDR was applied to control for multiple tests, especially with reference to inter-omics correlations, with a significance threshold FDR < 0.05 [40].

Bahado-Singh et al. [41] tested the application of different AI models, including DL, RF, SVM, PAM, linear discriminant analysis and generalised linear model (logistic regression) to amniotic fluid metabolomics and proteomics, alone and in combination with ultrasound, clinical and demographic factors, in predicting perinatal outcome in asymptomatic pregnant women with shortened cervical length. The results showed that each individual ML technique provided statistically significant predictions of different perinatal outcomes, but DL showed the best performance. The correction method for multiple tests performed is not explicitly specified in the study [41].

To date, academic research is driven by the important concept of open science [11,12]. However, the publication of the results obtained from analysed studies in public data repositories is still not an automatic procedure and very often it is complex to transform raw datasets into formats suitable for sharing as required by the most important public repositories. Indeed, the literature review shows that sometimes it is not explicitly stated whether the results are available in public scientific repositories [33,39,41] or it is made explicit that the results have to be requested from the authors [31,35,38]. Only in some cases are results uploaded on GitHub repositories [26] and sometimes on major metabolomics platforms, such as Metabolomic Workbench [27,40].

Table 2 summarises the potential biomarkers and/or the most relevant molecular pathways involved in common obstetric syndromes revealed by the combined analysis of metabolomics plus at least one other omics technology.

### 3.2. Newborns

The use of multiomics studies and precision medicine has begun to involve the neonatal field as well, especially in cases of prematurity, a condition in which diagnostic timing can be crucial. The study of the premature infant’s microbiota has been the subject of a metabolomic-focused multiomics approach [42,43], given its centrality in some devastating pathologies of the preterm infant, such as necrotising enterocolitis (NEC) or late onset sepsis (LOS) [44,45,46,47,48,49,50], conditions characterised by complex diagnostic pictures that often invalidate diagnostic timelines [9]. Two other neonatal pathological conditions that have been investigated using a metabolomic-focused multiomics approach are neonatal respiratory distress syndrome (NRDS) [51] and white matter injury (WMI) [52].

Multiomics studies on major neonatal problems that integrated metabolomic data with one or more other omics data are summarized in Table 3, where the type of correlation analysis between the different omics, if present in the studies, was highlighted.

Orchanian et al. [42] conducted an integrated metabolomics and microbiomics study to analyse the development of gut, skin, and oral microbiomes of preterm infants for the first week of life. Daily profiling of the first week after birth showed that the skin microbiome is highly resistant to early perturbation, while direct exposure of infants to antibiotics, rather than presumed maternal transmission, delays microbiome development and prevents early differentiation by body site, regardless of mode of delivery. Instead, metabolomic analyses showed a common developmental trend of all gut metabolomes of preterm infants toward the profiles of term infants. However, a significant increase in primary bile acid metabolism, with a transition from primary (non-microbe-modified) to secondary (microbe-modified) bile acids, occurs only in late-preterm infants not treated with antibiotics and for those born vaginally. A comparison, conducted by dividing the data by day of infant life, antibiotic exposure and mode of delivery, between metabolomic and microbiomic data by the Mantel test, showed no significance for the first four days after birth, while on days 5, 6 and 7 it was significant in late-preterm infants born by vaginal delivery never exposed to antibiotics. In addition, the microbial community and metabolome, showed significant correlations in all groups of late-preterm infants except those born by caesarean section and exposed to antibiotics, supporting an important role of microbiota on the child’s metabolic development. These findings can be considered a baseline for future multiomics and multi-site analyses of these populations of high-risk preterm infants with a view to providing new opportunities for monitoring and intervention. No correction for multiple testing was applied. [42].

Young et al. [43] conducted a nested cohort study within a randomised controlled trial (RCT) with the aim of evaluating the impact of bovine lactoferrin supplementation on the gut microbiome and metabolome of preterm infants. Therefore, a longitudinal analysis of the gut microbiome and metabolome of faeces and urine was conducted together with the analysis of volatile organic compounds (VOCs). The microbiome analysis showed no significant difference at 34 weeks of corrected age between infants in the two groups (lactoferrin and placebo). In fact, lactoferrin intake explained less than 1% of the variance in microbiome composition between the groups, in contrast to the impact of hospital site (16%) and postnatal age (6%). Metabolomic analyses also showed a limited impact of supplement intake. However, despite the lack of overall impact of supplementation, the authors conducted a multivariable association of the microbiome with linear models (MaAsLin2) to test for the possible presence of characteristics that differed as a function of lactoferrin receipt not only within the individual NICU but also among all the different NICUs. From the combined analysis of each NICU site, taking into account all clinical factors, the proportional abundance of only three unidentified stool metabolites and acetic acid was significantly higher in infants receiving lactoferrin, while a single stool metabolite, also unidentified, together with 2-methyl-propanal were significantly higher in placebo. In contrast, with regard to the microbiome, no bacterial genus remained significantly associated with either trial arm. FDR control was performed, with multiple test correction, using the Benjamini–Hochberg procedure [43].

Closely related to the microbiota of the premature infant is NEC; in fact, the alterations in the gut microbiota appear to play a key role in the genesis of this pathology. Indeed, the use of “omics” technologies, especially metabolomics and microbiomics, is becoming more widespread in the study of the pathophysiology of NEC in order to identify potential biomarkers to optimise diagnostic timelines and provide appropriate treatment [6]. In this regard, to date, only six metabolomics studies of NEC have integrated analysis with another omics technology, almost always microbiomics [44,45,46,47,48] but also proteomics, by always analysing a small number of samples [49].

Morrow et al. [44] from 16S RNA and urinary metabolome analysis of a very small number of infants, observed two distinct forms of gut microbial alteration that preceded all cases of NEC, with obvious repercussions on the metabolomic profile. In fact, through correlation between microbiomics and metabolomics (Kruskal–Wallis test and Spearman analysis), it was possible to find that changes in urinary metabolites correlated closely with the specific dysbiosis preceding disease development. Specifically, alanine was positively associated with cases of NEC preceded by *Firmicutes* dysbiosis and histidine was inversely associated with cases of NEC preceded by *Proteobacteria* dysbiosis. However, only a high urinary alanine:histidine ratio was found to provide a good prediction of overall NEC, thus representing a potential biomarker. Also in this study FDR control for multiple testing was performed with FDR significance threshold < 0.05, using the Benjamini–Hochberg procedure [44].

Work conducted by Stewart et al. [45] also highlighted the need for a multiomics approach, at least based on metabolomics and microbiomics, in identifying robust biomarkers for NEC. Albeit in a small number of samples, they conducted detailed temporal profiling of both bacterial and metabolomic gut microbiomes over the course of NEC. They found the presence of five discriminatory metabolites in NEC samples, related to biosynthesis of steroid hormones C21, linoleic acid (two different metabolites involved) and leukotrienes metabolism and formation of prostaglandins (from arachidonic acid), despite the absence of a unique microbial signature in NEC. Furthermore, metabolomics and 16S RNA sequencing data were highly concordant, with the NEC-associated metabolites significantly reduced in the healthy microbiome cluster. Moreover, given the increased intensity of these alterations before diagnosis and a decrease afterwards, they led the authors to hypothesise a good predictive potential for disease onset, with the possibility of 1–2 weeks earlier diagnosis than the current clinical diagnosis. The study explicitly states that the correction for multiple tests was carried out using the FDR algorithm. However, the significance threshold applied after correction is not explicitly specified [45].

Confirmation of the presence of a specific faecal metabolomic signature in NEC cases, along with alterations in the microbiota, also comes from the study by Brehin et al. [46]. They investigated the gut microbiota and metabolome of children with suspected NEC (NEC-1), showing specific alterations in the gut microbiota associated with NEC (abundance of *Streptococcus* species, second 10 days of life and *Staphylococcus* species, third 10 days of life) together with a different metabolomic signature than in healthy children. Specifically, no significant metabolomic changes emerged in the first 10 days of life, whereas a significant reduction in serine levels was detected in the second 10 days (11–20 days) along with lower concentrations of ethanol and leucine in the second month of life. This also allowed the third 10 days of life to be identified as a precise time window for intervention to combat NEC-1 progression. Correction for multiple testing was performed using the two-stage procedure of Benjamini, Krieger and Yekutieli, which corrects for multiple comparisons by checking the FDR with a significance threshold of less than 0.05 [46].

Recent work by Du et al. [47], through a targeted metabolomic analysis of stool together with a 16S RNA analysis of the faecal microbiota, also revealed the usefulness of a multiomics approach in the search for potential biomarkers for early diagnosis of NEC. The results of the study show that the decrease in unclassified *Staphylococcus*, *Lactobacillaceae* and *Bifidobacterium animalis subsp. lactis* at the species level together with the increase in the content of some tricarboxylic acid metabolites (succinate, L-malic acid and oxaloacetate) may represent possible early biomarkers of the disease. Correlation analysis (Spearman analysis) also showed interdependence between metabolome and faecal microbiota changes detected in the study, especially for two metabolites, L-malic acid and oxaloacetate. No explicit mention in the text of the multiple tests correction [47].

Another condition associated with high morbidity and mortality in preterm infants is LOS, and as in the case of NEC, gut microflora appears to play a crucial role. Also, at this juncture, research has focused on identifying early biomarkers to make early and differential diagnoses with other similar conditions, such as NEC. In fact, the two conditions are characterised by a significant overlap of nonspecific clinical signs in the absence of sensitive and specific diagnostic tools, conditions that make rapid, reliable and differential diagnosis very difficult [53].

In this regard, Stewart et al. [48] conducted bacterial profiling by 16S RNA sequencing together with a metabolomic analysis of stool on LOS infants. Microbiota analysis revealed fewer preterm intestinal community types (PGCTs) in LOS infants, unlike healthy controls, which were dominated by *Bifidobacteria*. Regarding the faecal metabolome, the observed changes were significant at diagnosis and 7 days later, but not in the 7 days prior to diagnosis. Specifically, 14 faecal metabolites were significantly altered between LOS and control infants at diagnosis; however, after correction for confounding factors, an increase of only seven metabolites in controls remained significant. Among these, raffinose, sucrose and acetic acid are closely related to the gut microbiota, while 18-hydroxycortisol, L-glutamate and 18-oxocortisol could be related to metabolic stress or inflammation responses. The results of correlation analysis through empirical correlation analysis software MixOmic (through sPLS discriminant analysis) showed clear interdependencies between the significant metabolites and the abundance of some bacterial genera in the pathology. Specifically, the abundance of *Bifidobacterium* correlated positively with the increase of certain metabolites (especially raffinose and acetic acid) in controls, supporting the importance, for gut development and protection in preterm infants, of certain prebiotic oligosaccharides (such as raffinose) and the growth of beneficial bacteria. Other correlations with altered metabolites have emerged for *Streptococcus* and *Morganella.* In the study, correction for multiple tests is performed using the Benjamini–Hochberg algorithm to control the FDR in both microbiome and metabolome analyses. However, the significance threshold applied after correction is not explicitly stated [48].

In order to optimise the differential diagnosis with NEC, Wandro et al. [49] performed bacterial composition characterisation by 16S rRNA gene sequencing and untargeted metabolomics analysis of stools from preterm infants with very low birth weight in the first 6 weeks of life in infants with NEC, LOS and healthy controls. However, the results of this analysis showed that no metabolites were characteristic for NEC or LOS and that the gut microbial communities of preterm infants were individualised, although they reflected antibiotic use. Nevertheless, correlation analysis between bacterial compositions of samples and metabolite profiles conducted with the Mantel test found a weak but significant association between bacterial composition and metabolite profile in preterm infants. No explicit mention in the text of the multiple testing correction [49].

The absence of specific biomarkers for NEC and LOS also emerged from the study by Stewart et al. [50]. They conducted a multiomics study by longitudinal analysis of the metabolome and serum proteome in preterm infants with these conditions. The results showed that in no case of NEC or LOS was a single protein or metabolite detected that was absent in controls, although several proteins (eight for NEC and four for LOS) associated with the disease state were observed, probably due to the different pathophysiology of the two conditions. Also, in this study, no explicit mention was made in the text of the correction for multiple testing [50].

NRDS, another critical condition in preterm infants due to immature lung development, has recently been studied using a multiomics approach. Indeed, Bi et al. [51] conducted a serum metabolome survey and longitudinal 16S RNA sequencing (weekly sampling for one month) of the gut microbiota in NRDS. The results revealed marked impairment of tryptophan metabolism, alterations in cortisol metabolism (high levels of intermediates, such as pregnenolone and 17α-hydroxyprogesterone) and accumulation of medium- and long-chain fatty acids (probable association with mitochondrial dysfunction observed) in the NRDS group, along with high relative abundance of *Haemophilus*, *Fusicatenibacter* and *Vibrio*. However, sequential microbial profiling suggested a distinct evolution of the gut microbiota in subjects with NRDS, characterised by a general reduction of potentially pathogenic bacteria (*Fusicatenibacter*, *Parabacteroides*, and *Holdemanella)* compared with other preterm infants. The authors also pointed out that these genera showed a sharp decline in abundance during the first week after birth, consistent with the typically short acute phase of NRDS. In contrast, integrated multiomics analysis conducted with empirical correlation analysis software MixOmic showed an inverse relationship between tryptophan concentrations and abundance of the anaerobic genus *Blautia*. Correction of the multiple testing was performed with the FDR method at a significance level of *p* < 0.05 [51].

A further application of multiomics investigation was conducted in very low or extremely low birth weight preterm infants (VLBW/ELBW) with WMI. Liu et al. [52] analysed mediated 16S RNA sequencing and liquid chromatography with tandem mass spectrometry (LC-MS-MS) the gut microbiota and faecal metabolome of preterm neonates with gestational age < 32 weeks and weight < 1.5 kg. After brain scanning by MRI at a corrected gestational age of 37–40 weeks, the infants were divided into two groups: 23 WMI and 48 non-WMI. 16S RNA sequencing showed severe dysbiosis of the gut microbiota in the WMI group, while metabolomic analysis showed a significant difference in the expression of 139 metabolic markers between WMI and non-WMI. KEGG pathway enrichment analysis showed that the WMI group had significant downregulation of 17 metabolic pathways, including arginine biosynthesis and primary bile acids. Delayed brain myelination, especially in the paraventricular white matter and splenium of the corpus callosum, was observed in the WMI group. Multiomics correlation analysis (Spearman analysis) showed that some groups characteristic of WMI-related dysbiosis, such as *Bacteroidetes*, *Staphylococcus*, and *Acinetobacter*, are strongly associated with alterations in the faecal metabolome (down-regulation of colic and allocholine acid and up-regulation of cinobufagin, adenosine-3-monophosphate and N-acetylneuramic acid), leading the authors to hypothesise a causal link with the damage found in the white matter of the brain, potentially attributable to the reduction of the bile acid biosynthesis pathway. Also in this study, no explicit mention was made in the text of the correction for multiple testing [52].

Also, in multiomic studies on newborns, sometimes it is not explicitly stated whether the results are available in public scientific archives [46]. Others place them in National Center for Biotechnology Information (NCBI) repositories [44,47] or GitHub repositories [48] and sometimes on major metabolomics platforms, such as Metabolights [45].

Table 4 summarises the potential biomarkers and/or the most relevant molecular pathways involved in major neonatal disorders revealed by the combined analysis of metabolomics plus at least one other omics technology.

## 4. Conclusions

The world of medicine is close to an epochal change, generated by the merging of the systems biology paradigm, which integrates information of different natures in an attempt to study the network of dynamic connections existing between genes, proteins, metabolites, and modern data analysis technologies. In this context, metabolomics plays a pivotal role, as it captures the direct biochemical response to a stimulus by detecting alterations in metabolites, the downstream end products of gene transcription and their perturbations. This enables the creation of a comprehensive snapshot of the phenotype of the studied organism. However, only integrating metabolomics with other omics can provide elucidation of the complex interactions occurring within a biological system. The application of AI further enhances this approach by generating insights and allowing the system to reason and learn from large, complex datasets. This synergy lays the foundation for precision medicine, which aims to optimise care for patients with less common responses to treatment or unique health needs.

The benefits of this approach in perinatology are innumerable, enabling optimisation of physician decision making through augmented intelligence that can combine non-genomic and genomic determinants, symptomatology, medical history and patient lifestyle.

The literature review showed that several perinatal medical conditions have begun to be explored through multiomics studies. Although still in their early stages, these studies suggest that many other complex pathological conditions could benefit greatly from this approach. For instance, the identification of biomarkers for detecting perinatally relevant pathological conditions, such as hypoxic–ischemic encephalopathy (HIE) secondary to perinatal asphyxia, earlier and more accurately, could optimise their clinical management. Additionally, a better understanding of the pathogenetic basis of certain critical clinical conditions, such as bronchopulmonary dysplasia, could lead to improving the therapeutic strategies in use today, potentially incorporating preventive approaches based on the first multiomics data on neonatal respiratory distress syndrome (NRDS).

However, for the synergy between multiomics studies and AI to translate into clinical relevance, it will be necessary to adopt unified data formats in sufficient quantity and quality for adequate algorithm training. A crucial step in this process is the sharing of data via major public data repositories. Another key challenge is the economic issue, given the high cost, to date, of multiomics analysis.

Moreover, the vast amounts of data generated by modern high-throughput technologies require integration that is complex, not solely due to the vast quantity of information, but also in view of the paucity of universal protocols. It is precisely the lack of universal protocols that causes difficulties in reproducibility and compliance with clinical standards, which then also poses a challenge to generalising AI models among different clinical centres.

Although the integration and translation of multiomics data into clinically relevant data is still a major hurdle, the use of multiomics analysis in biomedical research is becoming more widespread. This fits well with the absolute need to improve the preventive strategies available to date, especially in very vulnerable life epochs, such as the neonatal period, which represent crucial windows of opportunity for intervention. Moreover, this not only allows optimisation of diagnostic timelines and prognoses, facilitating careful therapeutic monitoring (crucial conditions in perinatology), but also the endotyping of diseases. This, in turn, supports the development of highly personalised treatments, which are particularly crucial for fragile neonatal patients.

Ultimately, only when the computational analysis of biological and clinical datasets is fully integrated into clinical practice can the goals of precision medicine be truly achieved.

## Figures and Tables

**Table 1:** Pregnancy’s multiomics metabolomics-focused studies.

Author and Year	Participants	Samples	Study Design	Omics	Main Results	Multiomics Correlation Analysys
Gong et al. (2018) [25]	4212 nulliparous women, 134 PE, 162 IUGR e 259 controls	Placental samples, serum	Prospective cohort study	Placental transcriptome, placental methylome Mother’s serum metabolome	Different placental polyamine metabolism by fetal sex	No correlation analysis (multiomic approach to identify polyamine synthesis pathway implicated in the pathophysiology of placentally related com- plications of human pregnancy)
Barack et al. (2023) [26]	75 severe PE 40 IUGR 33 IUGR + HDP 72 sPTD 2 control groups: −113 term −16 preterm without PE, IUGR or sPTD	Placental samples	Retrospective cohort study	Transcriptomic, proteomic, metabolomics (biobank)	Omics-based placental dysfunctions clustering showed a better correlation with histopathology than the predefined disease clusters and could improve diagnostic accuracy	Unbiased AI-based SNF
Marić et al. (2022) [27]	33 women in the discovery cohort (17 PE, 16 normotensive) 16 women in the validation cohort (12 PE, 4 normotensive)	Plasma, vaginal swab, urine	Longitudinal, prospective cohort study	Six omics datasets cell-free RNA (cfRNA)/transcriptome, proteome, me- tabolome, lipidome and microbiome	Prediction models from urine metabolome and from proteome had the best accuracy	ML (Elastic net)
Kelly et al. [28]	47 pregnant women from VDAART cohort who developed PE and 62 healthy controls	Plasma	Prospective cohort study	Metabolomics and transcriptomic	Predictive PE serum metabolites from the first trimester of pregnancy with moderate to good discriminatory ability in the third trimester Integration with transcriptomic data provided a deeper understanding of the biology underlying PE	Correlation clustering analysis (WGCNA)
Odenkirk et al. (2020) [29]	191 women (98 controls, 45 GDM, 48 PE)	Plasma (Peribank)	Case control study	Proteomics and lipidomics	Direct relationship between proteins and lipids, suggesting a strong interdependence in the origin and progression of GDM and PE	Multi-omic assay/comparison
Easton et al. (2023) [30]	-	BeWo cell line model	In vitro model + Multiomics approach	Transcriptomic (mRNA microarray), metabolomic, and lipidomic	↑ certain dietary fats in maternal circulation involved in facilitating the aberrant placental function in obese and GDM pregnancies Saturated and unsaturated FA species differentially impact placental function	Pathways enrichement analysis software (MetaboAnalyst v5)
Dong et al. (2020) [31]	40 pregnant women (20 GDM and 20 non-diabetic control)	Plasma, faecal samples	Case control study	Metabolomics and microbiomics	Alterations in faecal microbiota associated with hyperglicemia-related changes of the plasma metabolome of GDM women suggesting novel therapies against gut microbiota to alleviate GDM	Correlation analysis (co-inertia, procrustes and redundancy analysis)
Liu et al. (2024) [32]	89 pregnant women and their neonates (44 with GDM, 45 controls)	Maternal faecal and blood samples, umbilical cord blood and neonatal meconium	Prospective cohort study	Metabolomics and microbiomics	Distinct microbial and metabolic signatures in mothers with GDM and their infants Metabolites related to glucose, lipid and energy metabolism were affected differently in GDM, underlying metabolic transmission across generations	Correlation analysis (Spearman, R packages corrplot, ggalluvial, and ggplot)
Fu et al. (2024) [33]	750, 748 and 709 pregnant women had ITS2 sequencing data, 16S sequencing data and serum metabolome data available, respectively, across all trimesters.	Stool samples and blood samples	Cohort study	Metabolomics, microbiomics and mycobiomics	Significant interactions between the intestinal mycobiome, biological function, serum metabolites and the course of pregnancy Correlation between the presence of Mucor and the onset of GDM /fetal macrosomia Pre-pregnancy overweight influences both altered intestinal mycobiome composition and metabolic remodelling during pregnancy	Correlation analysis (regression, network and Spearman analysis)
Tao et al. (2023) [34]	70 pregnant women (35 IUGR, 35 healthy controls)	70 faecal samples, 50 serum samples (31 from controls group, 19 from IUGR group)	Cohort study	Metabolomics and microbiomics	Intestinal dysbiosis and metabolic disorders in IUGR patients, which contribute to the pathogenesis of the disease Both microbial species and altered metabolites in IUGR are closely related to fetal measures and maternal clinical variables	Correlation analysis (Spearman)
Tang et al. (2024) [35]	20 pregnant women (11 healthy pregnant women, 9 IUGR)	Umbilical cord blood, maternal serum, feces, placental tissue samples	Cohort study	Metabolomics, transcrptomics and microbiomics	Metabolomic, microbiomic and transcriptomic alterations mediate the interaction between the gut and the circulatory system in IUGR.	Correlation analysis (Spearman)
Jehan et al. (2020) [36]	81 pregnant women (39 PTD, 42 term pregnancies) from 5 different regions	Urine, plasma	Prospective cohort study	Metabolomics, transcriptomics and proteomics	An inflammatory component (from transcriptomics and proteomics data) and specific alterations in the urinary metabolome were the main PTD associated features. Predictive accuracy for PTD increases due to a multiomics approach	Higher-level ML
Espinosa et al. (2023) [37]	231 pregnant women: (113 PTD, 118 with a term delivery)	Blood samples	Cohort study	Metabolomics, lipidomics and proteomics	ML models showed robust performance for the prediction of PTD, time-to-delivery, maternal age, gravidity and BMI	ML
Wu et al. (2024) [38]	35 pregnant women (13 normal pregnancy but indicated for caesarian section because of a scarred uterus, 22 pathological pregnancy)	Peripheral blood, umbilical cord blood and placenta samples	Observational and comparative study	Metabolomics, lipidomics and transciptomics	Aberrant immune activation and an abnormal increase of lipids and lipid-like metabolites in the placental microenvironment in PTD The proportion and activation of MAIT cells were positively correlated with the abnormal increase in lipids and lipid-like metabolites	Correlation analysis
Ghaemi et al., 2019 [39]	17 pregnant women, delivering at term	Plasma and serum samples, whole blood samples and vaginal swabs, stool, saliva and tooth/gum samples	Prospective cohort study	Metabolomics, immunomics transcriptomic, microbiomics and proteomics	A singlle model created from 7 high-throughput longitudinal biological assays of the same patient cohort showed both significantly greater predictive power of gestational age and new interactions between different biological modalities	ML (stacked generalization algorithm)
Stelzer et al. (2021) [40]	63 healthy pregnant women with spontaneous labour (of which 5 preterm)	Serial blood samples collected during the last 100 days of pregnancy	Longitudinal observational study	Metabolomics, proteomics and immunomics	Co-ordinated alterations in the metabolome, proteome and maternal immunome marked a molecular transition from pregnancy maintenance to pre-birth biology 2 to 4 weeks before delivery	ML (stacked generalization algorithm)
Bahado-Singh et al. (2019) [41]	26 pregnant women asymptomatic with short CL (<15 mm) of these, 11 patients delivered ≥ 34 weeks, while 15 delivered < 34 weeks	Amniotic fluid	Retrospective observational study	Metabolomics and proteomics	Good to excellent prediction of perinatal outcome in asymptomatic pregnant women with short CL in the second trimester with combined omics, demographic and clinical data, machine learning, particularly deep learning	DL, RF, SVM, generalized linear model, PAM, LDA

Abbreviations: PE, preeclampis; HDP, hypertensive disorder; IUGR, intrauterine growth restriction; sPTD, spontaneous preterm delivery; PTD, preterm delivery; SNF, similarity network fusion; AI, artificial intelligence; ML, machine learning; DL, deep learning; SVM, support vector machine; PAM, prediction analysis for microarrays; LDA, linear discriminant analysis; WGCNA, weighted gene correlation analysis; CL, cervix length.

**Table 2:** Common obstetric syndromes potential biomarkers and/or most relevant molecular pathways.

Clinical Condition	Possible Biomarkers or Main Involved Pathways	Multiple Testing Correction Method	Authors
**PE-IUGR**	DiAcSpm is the first circulating maternal metabolites demonstrating opposite associations with PE and IUGR (higher maternal serum levels of DiAcSpm associated with ↑ PE risk but ↓ IUGR risk)	No FDR correction but unadjusted *p*-value < 0.01 for metabolites FDR-adjusted *p*-value < 0.05 for other analysis (Benjamini–Hochberg procedure)	Gong et al. [25]
	Significantly altered metabolites included mainly lipids and amino acids: ↑ sphingolipid species associated with IUGR and HDP involvement in interleukin signaling, interferon and glucose metabolism	FDR-adjusted *p*-value < 0.05 (Benjamini–Hochberg procedure) for metabolic pathway enrichment analysis and canonical pathways	Barak et al. [26]
**PE**	Lipid and amino acid metabolism changes: metabolism of glycerophospholipids, fatty acids and arachidonic acid metabolic imbalances related to fatty acid synthesis pathways regulation of immune function and circulatory system	No correction for multiple testing Some metabolites and biological pathways were confirmed through replication between first- and third-trimester samples	Kelly et al. [28]
	Tryptophan metabolism alteration involving kynurenine pathways Fatty acid and arachidonic acid metabolism changes together with steroid hormone metabolism alteration	FDR-adjusted *p*-value < 0.05 (Benjamini–Hochberg procedure)	Marić et al. [27]
**IUGR**	Possibile mediators in the interaction between the gut and the circulatory system in IUGR: altered metabolites (including methionine and alanine) changes in some microbial species (Tyzzerella) alteration of circulating mRNAs (TRIM34, SMOX, FAM83A, NAPG)	No correction for multiple testing Unadjusted *p*-value < 0.05 in Spearman’s correlation analysis	Tang et al. [35]
	Significant association between 25 major bacterial species and 13 serum metabolites (allantoin, pinitol, nicotinic acid, malic acid, phosphatidic lysoacid, maltose, 9-hexa-decene, glycol-diacid-2-phosphate) together with 4 of faecal origin (dodecanoic glycol, dodecanoic glycol IV and pyrraline) Both faecal (physangulin E, Ginkgolide C and pyrraline) and serum metabolites (allantoin and malic acid) altered in IUGR were also found to be associated with specific clinical phenotypes (multiple fetal measures and maternal biochemical markers)	FDR adjusted *p*-value thresholds: <0.05, <0.01, <0.001 for microbiota analysis and <0.001 for differences in serum metabolites	Tao et al. [34]
**GDM**	Correlation between the faecal microbiota and the plasma metabolome in GDM related to five plasma metabolites (↑ lactic acid, proline, galactitol and ↓ glycerolmethylmalonic acid) and 98 members of the faecal microbiota (mainly members of the Firmicutes)	FDR-adjusted *p*-value < 0.05	Dong et al. [31]
	Alteration in bile acid metabolism, certain lipid metabolites related to the metabolic pathway of unsaturated fatty acids and lipid biosynthesis in both mothers and diabetic offspring: ↓ glycerophospholipids (e.g., PC(18:0/20:3(5Z,8Z,11Z)) in both mothers and infants with GDM ↓ polyunsaturated fatty acids (e.g., PE (18:4/22:6)) in diabetic mothers but ↑ GDM infants	FDR-adjusted *p*-value < 0.05 (Benjamini–Hochberg procedure)	Liu et al. [32]
**PE-GDM**	PE group: ↑ coagulation protein and complement cascade protein ↓ phosphatidylinositol GDM group: ↑ phosphatidylethanolamine plasmanogel species ↓ phosphatidylethanolamine-binding protein 1 (PEBP1)	ANOVA with Dunnett correction G-test with Bonferroni correction FDR-adjusted *p*-value < 0.05	Odenkirk et al. [29]
**PTD**	Specific alterations in the PTD urinary metabolome: glutamine and glutamate metabolism valine, leucine and isoleucine biosynthesis pathways From proteomic and transcriptomic data: ↑ inflammatory pathways	Fisher’s tests for enrichment of metabolic pathways: unadjusted *p*-value < 4.4 × 10^−^⁹ for glutamine and glutamate metabolism, unadjusted *p*-value < 7.3 × 10^−6^ for valine, leucine and isoleucine biosynthesis Bonferroni-adjusted *p*-values of < 0.05 (transcrittomic analysis) FDR-adjusted *p*-value < 1.0 × 10^−6^ (proteomic analysis but some tests report incorrect *p* values as more specific or limited comparisons)	Jehan et al. [36]
	Specific fetal proteins (e.g., ALPP, AFP, and PGF) and immune proteins (e.g., PD-L1, CCL28, and LIFR) showed correlation with time to delivery	Bonferroni-adjusted *p*-values of <0.05 and |Rho| > 0.38	Espinosa et al. [37]
	Aberrant immune activation and abnormal increase of lipids and lipid-like metabolites in the placental microenvironment Heatmap correlation analysis showed that placental MAIT cell activation and expression of pro-inflammatory tissue molecules were positively correlated with the levels of most elevated metabolites (sphingolipids, lysolipids, acylcarnitines)	No correction for multiple testing Unadjusted *p*-value < 0.05 for differences in metabolites	Wu et al. [38]
**Time of Delivery**	Plasma proteomics has the highest predictive power among the individual datasets Correlation between pregnanolone sulfate and immune system and between the oral microbiome and the TCRγδ⁺ cells	Bonferroni correction with significance threshold adjusted according to the number of comparisons made in the dataset	Ghaemi et al. [39]
	↑ in steroid hormone metabolites and interleukin-1 type 4 receptor before labour, coinciding with the shift from immune activation to regulation of inflammatory responses	FDR-adjusted *p*-value < 0.05	Stelzer et al. [40]

Abbreviations: ↑ increased; ↓ decreased; FDR, false discovery rate; PE, preeclampsia; HDP, hypertensive disorder; IUGR, intrauterine growth restriction; PTD, preterm delivery; GDM, gestational diabetes mellitus.

**Table 3:** Neonatal’s metabolomics-focused multiomics studies.

Author and Year	Participants	Samples	Study Design	Omics	Results	Multiomics Correlation Analysys
Orchanian et al. (2022) [42]	75 preterm infants: 29 LP-C, 28 LP-V, and 18 VLBW-C	gut, oral, and skin samples	Cohort study	Metabolomics and microbiomics	Skin microbiome is robust to early perturbations, whereas direct exposure of infants to antibiotics, rather than presumed maternal transmission, delays microbiome development and prevents early differentiation according to body site, regardless of mode of delivery	Mantel test
Young et al. (2023) [43]	479 infants born <32 weeks (gut microbiome 201 infants, metabolites 83 infants for stools and 90 for urine, VOCs 117 infants)	Fecal and urine samples	Nested cohort study (within a randomised control trial)	Metabolomics and microbiomics	Minimal impacts of lactoferrin but much larger impacts of hospital site and postnatal age on the gut microbiome and metabolome of preterm infants	No correlation analysis
Morrow et al. (2013) [44]	11 NEC vs. 21 controls	Fecal and urine samples	Prospective observational study	Metabolomics and microbiomics	Urinary metabolites variations are closely related to the specific dysbiosis preceding disease development	Correlation analysis (Spearman analysis, Kruskal–Wallis test)
Stewart et al. (2016) [45]	7 NEC vs. 28 controls (of which 6 NEC and 10 controls also performed metabolomic analysis)	Stool samples	Cohort study	Metabolomics and microbiomics	Absence of a NEC uniform microbial signature but metabolomic profiling showed 5 discriminant metabolites in NEC samples	Correlation analysis
Brehin et al. (2020) [46]	11 NEC stage I vs. 21 controls	Fecal samples	Prospective cohort study	Metabolomics and microbiomics	Changes in the gut microbiota and microbiome in NEC-1 appear more evident from day 20 onwards, compared to healthy controls, identifying this precise time window as a therapeutic/diagnostic target for NEC	No correlation analysis
Du et al. (2023) [47]	16 NEC vs. 16 non NEC controls	Fecal samples	Prospective cohort study	Metabolomics and microbiomics	Interdependence between metabolome and faecal microbiota changes Potential value for NEC early diagnosis for ↓ unclassified microbiota species as well as ↑ TCA metabolites	Correlation analysis (Spearman)
Stewart et al. (2017) [48]	7 LOS and 28 matched healthy (no LOS or NEC) controls	Fecal samples	Observational longitudinal cohort study	Metabolomics and microbiomics	Multi-omic analysis showed *Bifidobacterium* was positively correlated with metabolites significantly ↑ in control infants	Empirical correlation analysis software (MixOmics, through sPLS-DA)
Wandro et al. (2018) [49]	32 VLBW (3 NEC 8 LOS and 21 controls)	Fecal samples	Retrospective observational cohort study	Metabolomics and microbiomics	Bacterial abundances varied over 4 orders of magnitude and were ↓ in infants that developed NEC or LOS No metabolites or microbiome signature associated with NEC or LOS	Mantel test and Pearson correlation
Stewart et al. (2015) [50]	19 patients (6 NEC, 4 LOS vs. 9 controls)	Serum samples	Prospective cohort study	Metabolomics and proteomics	No single protein or metabolite was detected in all NEC or LOS cases, which was absent from controls Notably, the only child who died during the study had a unique proteomic and metabolomic profile	No correlation analysis
Bi et al. (2024) [51]	13 preterm infants with NRDS and 12 preterm without NRDS	Blood and faecal samples	Longitudinal cohort study	Metabolomics and microbiomics	Presence of specific metabolic and microbiota alterations associated with the NRDS Inverse relationship between tryptophan concentrations and the abundance of the anaerobic genus *Blautia* (from integrated multiomics analysis)	Empirical correlation analysis software (MixOmic version 6.0.0.)
Liu et al. (2023) [52]	23 preterm with and 48 patients without WMI (WMI group included 12 cases of mild WMI, 7 cases of moderate WMI, and 4 cases of severe WMI)	Meconium (within 24 h after birth) and stool samples	Prospective cohort study	Metabolomics and microbiomics + MRI and DTI	Significant difference in the expression of 139 metabolic markers and severe gut dysbiosis in the WMI group Certain groups characteristic of WMI-related dysbiosis strongly associated with alterations in the faecal metabolome (from multiomics correlation analysis), with possible causal links to the damage found in the white matter of the brain	Correlation analysis (Spearman)

Abbreviations: LP-C, late preterm infants born via caesarean section; LP-V, late preterm infants born vaginally; VLBW-C, very low birth weight preterm infants born via caesarean section; VOCs, volatile organic compounds; NRDS, neonatal respiratory distress syndrome; WMI, white matter injury; MRI, magnetic resonance image; DTI, diffusion tensor imaging; MaAsLin2, microbiome multivariable association with linear models; sPLS-DA, spares partial least squared discriminant analysis.

**Table 4:** Major neonatal disorders potential biomarkers and/or the most relevant molecular pathways involved.

Neonatal Disorders	Possible Biomarkers or Main Involved Pathways	Multiple Testing Correction Method	Authors
**Prematurity**	Metabolomic analyses showed a common developmental trend of all gut metabolomes of preterm infants towards the profiles of term infants significant ↑ in primary bile acid metabolism occurs only in late-preterm infants not treated with antibiotics and born vaginally	No correction for multiple testing	Orchanian et al. [42]
**NEC**	only high urinary alanine:histidine ratios provide a good prediction of overall NEC, thus representing a potential biomarker alanine positively associated with NEC cases preceded by *Firmicutes* dysbiosis histidine inversely associated with NEC cases preceded by *Proteobacteria* dysbiosis	FDR-adjusted *p*-value < 0.05 (Benjamini–Hochberg procedure)	Morrow et al. [44]
	5 discriminant metabolites in NEC samples involved into C-21 steroid hormones, linoleate, prostaglandines, leucotrienes pathways with increased intensity before diagnosis and decrease afterwards	FDR-adjusted *p*-value applied but threshold not specified	Stewart et al. [45]
	The faecal metabolome of NEC-1 was more divergent from the second month of life the first changes were evidenced with a significant ↓ in serine levels in the second 10 days of life (11–20 days) ending with ↓ ethanol and leucine concentrations in the second month of life	Two-stage linear step-up procedure of Benjamini, Krieger and Yekutieli Significance threshold: *p* < 0.05	Brehin et al. [46]
	↓ in unclassified *Staphylococcus*, *Lactobacillaceae* and *Bifidobacterium animalis subsp. lactis* at the species level together with ↑ in the content of some TCA metabolites (succinate, L-malic acid and oxaloacetate) may represent possible early biomarkers of the disease interdependence between metabolome and faecal microbiota changes especially for two metabolites, L-malic acid and oxaloacetate	No correction for multiple testing	Du et al. [47]
**NEC + LOS**	No metabolites or microbiome signature associated with NEC or LOS	No correction for multiple testing	Wandro et al. [49]
	No discriminating metabolomic and proteomic biomarkers for NEC or LOS	No correction for multiple testing	Stewart et al. [50]
**LOS**	Changes in the faecal metabolome were significant at diagnosis and 7 days later, but not in the 7 days prior to diagnosis. 7 faecal metabolites were significantly altered between infants with LOS and control infants at diagnosis, after correction for confounding factors raffinose, sucrose and acetic acid were closely related to the gut microbiota, whereas 18-hydroxycortisol, L-glutamate and 18-oxocortisol could be related to metabolic stress or inflammatory responses.	FDR-adjusted *p*-value applied but threshold not specified (Benjamini–Hochberg procedure)	Stewart et al. [48]
**NRDS**	In NRDS group: marked impairment of tryptophan metabolism alterations in cortisol metabolism (↑ levels of intermediates, such as pregnenolone and 17α-hydroxyprogesterone) accumulation of medium and long-chain fatty acids (probable association with mitochondrial dysfunction) high relative abundance of *Haemophilus*, *Fusicatenibacter* and *Vibrio*	FDR-adjusted *p*-value < 0.05	Bi et al. [51]
**WMI**	Presence of characteristic groups in WMI-related dysbiosis (*Bacteroidetes*, *Staphylococcus* and *Acinetobacter*) strongly associated with alterations in the faecal metabolome (↓ colic and allocholine acid, ↑ cinobufagin, adenosine-3-monophosphate and N-acetylneuramic acid) mainly related to arginine and bile acid biosynthesis pathways	No correction for multiple testing	Liu et al. [52]

Abbreviation: ↑ increased; ↓ decreased; TCA, tricarboxylic acid; NEC, necrotising enterocolitis; LOS, late-onset sepsis; WMI, white matter injury; NRDS, neonatal respiratory distress syndrome.

## Data Availability

Not applicable.
